# Supplementary material for: From sequence to enzyme mechanism using multi-label machine learning
Source: BMC Bioinformatics. 2014 May 19;15:150. doi: 10.1186/1471-2105-15-150 (PMC4229970; doi:10.1186/1471-2105-15-150)
Supplement: Additional file 2 — Java code of ml2db. Additional file ml2db_code.tar.gz contains the Java source code to run the multi-label machine learning experiments and save the results to database. The code’s Javadoc is included. [file 1471-2105-15-150-S2.zip › additional file 2/ml2db/ml2db/doc/main/ml2db/al/utility/pickers/package-use.html]

Uses of Package main.ml2db.al.utility.pickers


JavaScript is disabled on your browser.


- Overview
- Package
- Class
- Use
- Tree
- Deprecated
- Index
- Help

- Prev
- Next

- Frames
- No Frames

- All Classes

# Uses of Package main.ml2db.al.utility.pickers

- Packages that use main.ml2db.al.utility.pickers

  | Package | Description |
  | --- | --- |
  |  |  |
  | --- | --- |
  | main.ml2db.al.utility.pickers |  |
  | main.ml2db.al.utility.pickers.byfrequency |  |
- Classes in main.ml2db.al.utility.pickers used by main.ml2db.al.utility.pickers

  Class and Description

  Picker

  A class that picks and returns an instance (random picking or ordering by
  frequency in the data etc.)

  RandomPicker

  Class
- Classes in main.ml2db.al.utility.pickers used by main.ml2db.al.utility.pickers.byfrequency

  Class and Description

  Picker

  A class that picks and returns an instance (random picking or ordering by
  frequency in the data etc.)

  RandomPicker

  Class

- Overview
- Package
- Class
- Use
- Tree
- Deprecated
- Index
- Help

- Prev
- Next

- Frames
- No Frames

- All Classes
